# Cultural tailoring of pain management approaches: a scoping review

**DOI:** 10.1186/s12939-025-02743-5

**Published:** 2025-12-20

**Authors:** Nourah Basalem, Anfal Adnan Astek, Roaa Abdulghani Sroge, Syed Mustafa Ali, Jaheeda Gangannagaripalli, Emma Stanmore, Sabine N. van der Veer

**Affiliations:** 1 Division of Informatics, Imaging and Data Science, Manchester Academic Health Science Centre, University of Manchester, Manchester, UK; 2 College of Applied Medical Sciences, Prince Sattam Bin Abdulaziz University, Al-Kharj, Saudi Arabia; 3 Faculty of Medical Rehabilitation Sciences, King Abdulaziz University, Jeddah, Saudi Arabia; 4 Faculty of Applied Medical Sciences, Umm Al-Qura University, Makkah, Saudi Arabia; 5 Healthy Ageing Research Group, School of Health Sciences, Faculty of Biology, Medicine and Health, Manchester Academic Health Science Centre, University of Manchester, Manchester, UK

**Keywords:** Cultural competency, Cultural tailoring, Ethnic and racial minorities, Health inequities, Pain management, Scoping review

## Abstract

**Background:**

Pain affects an estimated 1.5 billion people globally. Cultural factors strongly influence how pain is perceived, communicated, and managed. However, it remains unclear to what extent, how, and for whom pain management approaches have been culturally tailored, and whether these adaptations have been evaluated to ensure cultural relevance and effectiveness. This scoping review addresses this gap.

**Methods:**

We searched six electronic databases for peer-reviewed articles and grey literature, combining terms for pain and cultural tailoring. We included empirical studies (including protocols), published in English, that reported on the cultural tailoring of pain management approaches for adults (≥18 years). At least two reviewers independently screened titles and abstracts, followed by full text assessment. We charted data on study characteristics, cultural tailoring methods, and evaluation strategies, and synthesised results narratively.

**Results:**

Our search identified 4,551 unique studies, of which we included 38. Of these, 32 (84%) were published after 2016, with 26 (68%) focusing on musculoskeletal pain. They reported the cultural tailoring of 27 unique pain management intervention approaches, of which 19 (70%) focused on racial and ethnic minorities mainly in high-income countries. Educational interventions were most commonly tailored (*n* = 9, 33%). Only four (15%) tailored approaches were delivered digitally. Most (*n* = 25, 93%) approaches underwent content adaptation through including culturally relevant language, metaphors, and gender considerations. Most employed early tailoring steps, such as information gathering (85%) and preliminary adaptation design (93%). Only six (22%) approaches used frameworks to guide the adaptation such as Intervention Mapping-Adapt, FRAME, and ADAPT-IT. The effectiveness of cultural tailoring was evaluated for 11 (41%) approaches, mainly through randomised controlled trials (*n* = 7, 26%).

**Conclusion:**

This review identified several efforts to culturally tailor pain management approaches, particularly for racial and ethnic minorities with musculoskeletal pain in high-income countries. Most tailoring focused on content adaptation for in-person formats, with limited use of contextual modifications, digital delivery, or adaptation frameworks. Future research should broaden tailoring beyond content changes, make greater use of digital tools, and prioritise adaptations in low- and middle-income countries. Evaluation strategies should also expand to assess real-world implementation, and long-term outcomes.

**Supplementary Information:**

The online version contains supplementary material available at 10.1186/s12939-025-02743-5.

## Introduction

Pain is a complex and multifaceted phenomenon affecting an estimated 1.5 billion people worldwide. This represents a significant public health challenge that contributes to the global disease burden [1, 2]. Pain is more prevalent in some groups: women, older adults, ethnic minority groups, rural residents, and those who are unemployed or with lower income levels are more likely to experience pain [3–5]. These same groups also face wider health inequalities, as pain is closely linked to broader social determinants of health [6, 7].

Among these socioeconomic determinants, cultural factors also play a key role in the perception, communication, and management of pain. ‘Culture’, broadly defined as socially learned and group-specific behaviours, encompasses beliefs, values, and customs that impact a wide array of human behaviours, including responses to pain [8]. Beliefs about pain tolerance and expression vary widely; some cultures emphasize stoicism, leading individuals to underreport pain, whereas others encourage more open expression [9]. Similarly, treatment preferences differ across cultures, with some favouring pharmacological interventions and others opting for traditional remedies such as acupuncture or herbal treatments [10, 11]. Not acknowledging and addressing the influence of culture on pain reporting and treatment may hinder the uptake of and benefit from pain assessment and management approaches for some cultural groups, thereby perpetuating inequities in pain outcomes [12, 13].

Effective pain management is a collaborative process requiring engagement from both patients and healthcare professionals [14]. As patients’ cultural beliefs shape how pain is perceived and expressed, providers must adapt care to align with these values [15]. Many have advocated for recognising the influence of cultural factors in disease assessment and management approaches [16–19]. At the system level, this requires inclusive policies and infrastructures to support diverse populations [20, 21]. This includes culturally tailored approaches to deliver care in line with patients’ cultural contexts, enhance cultural competence of providers, and promote system-wide inclusivity [22] to ultimately improve health equity.

Beyond acknowledging cultural influences on care, several frameworks have been developed to guide the systematic adaptation of health interventions for diverse populations, balancing fidelity to evidence-based practices with cultural relevance. These frameworks commonly outline key components such as staged adaptation processes, stakeholder engagement, iterative refinement, and contextual evaluation [23–25]. They thereby offer a structured and evidence-informed foundation for achieving equitable, culturally responsive care. Cultural tailoring builds on this, as it often involves adapting evidence-based practices to better reflect the language, values, and norms of target populations [26]. In mental health research, culturally tailored disease management approaches have been extensively reviewed and documented [27–30], often incorporating not just translation, but also traditional healing practices, culturally relevant concepts, and the involvement of family or community members to improve engagement and outcomes [31, 32]. However, in the context of pain, several reviews showed that studies have primarily focused on cross-cultural tailoring of pain self-assessment tools and evaluating translation procedures and measurement properties [33–39]. It is currently unknown if and how cultural tailoring efforts have gone beyond self-assessment methods to include other elements of pain management. Our preliminary search of MEDLINE and the Cochrane Database of Systematic Reviews further suggested that there has been little exploration of the impact of cultural tailoring on the uptake and outcomes of pain management approaches.

This scoping review aimed to address these gaps by exploring the cultural tailoring of pain management approaches. Specific objectives were to (1): Identify and describe studies that culturally tailored pain management approaches (2); For studies identified under 1, describe (a) the approach that was tailored and the population for which it was tailored, and (b) what methods were used for tailoring aspects of the management approaches; and (3) If and how the impact of the cultural tailoring was evaluated. By providing insights into the extent and methods of cultural tailoring of current pain management approaches, we expect our review to guide and inform future cultural tailoring initiatives in this area.

## Methods

We conducted the scoping review to map the literature on cultural tailoring of pain management approaches beyond pain self-assessment tools, following the Joanna Briggs Institute methodology for scoping reviews [40], which builds on previous guidance developed by Arksey & O’Malley [41] and Levac et al. [42]. The Preferred Reporting Items for Systematic Reviews and Meta-Analyses extension for Scoping Reviews (PRISMA-ScR) [43] guided the reporting of this review (Supplementary Appendix 1).

## Review registration

The protocol [44] was registered with Open Science Framework on November 22, 2024.

## Eligibility criteria

Table 1 presents the eligibility criteria for study selection based on the Population, Concept and Context framework [40].

**Table 1:** Eligibility criteria for study selection

Criteria	Examples of inclusion	Examples of exclusion
**Population** *Adults aged 18 years or older with lived experience of pain or involved in pain management or pain management approaches*	- People experiencing pain of any type or in any bodily region. - People living with painful condition. - Any stakeholder involved in, developing or evaluating approaches to managing pain (healthcare professionals, tech developers, etc).	- Children with pain or their parents.
**Concept** *Cultural tailoring* Refers to adapting designs, materials, and components of disease management approaches to align with the cultural needs and preferences of a specific population. We also included studies who used related terms such as cultural competency, culturally appropriate, cultural adaptation, and cultural targeting [22], as long as they adhered to our definition.	- Studies to inform the cultural tailoring of management approaches - Studies developing an approach for a target population with a specific cultural background - Studies tailoring management approaches for populations different from or more specific than the original target population - Studies evaluating the uptake, use, or effectiveness of the tailored approach	- Studies not incorporating cultural considerations into management approach design. - Exploratory studies of cultural considerations without actionable recommendations for management approaches. - Studies on sociocultural factors influencing pain.
**Context** *Pain management* Encompasses a range of elements aimed at reducing pain and improving quality of life in any country or healthcare setting. This can include, for example, pharmacological treatments, non-pharmacological therapies, educational interventions, and self-management techniques [45].	- Integrating culturally informed approaches into physiotherapy assessment and treatment of chronic pain - Development of culturally sensitive pain neuroscience education materials for Hausa-speaking patients with chronic spinal pain	- Studies that reported solely on the cross-cultural tailoring of patient-reported questionnaires without embedding them in more comprehensive pain management programmes^1^
**Study Type**	- Empirical studies (qualitative, quantitative, mixed-methods) including protocols	- Systematic reviews, meta-analyses, commentaries, editorials, conference abstracts, or opinion pieces.
**Publication Type**	- Peer-reviewed journal and conference articles, theses and dissertations, and patient or professional organisations reports	Pre-prints
**Language**	- Full-text articles in English.	- Articles in languages other than English^2^ or without available full text.

^1^ We excluded studies solely focused on the cross-cultural adaptation of pain self-report tools because these had been extensively covered by previous reviews [33–39]

^2^ *Non-English articles were excluded due to lack of reliable resources to support translation*

## Search strategy

We searched three electronic bibliographic databases (EBSCO CINAHL, Ovid MEDLINE, and Ovid PsycINFO) between 13^th^-16^th^ of September 2024 for peer-reviewed studies and without restrictions based on publication date. For grey literature, we searched Google Scholar (first 800 titles) based on guidance from Haddaway et al. [46], as well as Global Health and UpToDate; the latter two did not yield any results.

Our search strategy combined key words and Medical Subject Headings terms for pain and cultural tailoring. For the concept of cultural tailoring, we selected the search terms used in previous reviews [24, 47]. Following a three-step process, we conducted an initial preliminary search of MEDLINE (Ovid) to identify relevant articles and extract keywords and index terms from the titles, abstracts, and descriptors. These terms formed the basis for developing a comprehensive search strategy that we refined and adapted for each database with guidance from an experienced librarian. Appendix 2 shows the full search syntax, which is also included in the supplementary materials of the registered protocol [44]. For Global Health, UpToDate, and Google scholar search terms were adapted from the main database strategy and tested and refined by screening the first two pages of Results for relevance. The syntax of the final grey literature search was: Pain AND (cultural adaptation OR cultural tailoring OR cultural consideration).

In the final step, and to complement our electronic search, we manually reviewed the reference lists of all included studies and relevant reviews.

## Study/Source of evidence selection

We used the online platform RAYYAN QRCI (https://rayyan.qcri.org/) [48] to import the search results, remove duplicates, and perform screening. Following a pilot test, at least two reviewers screened each title and abstract independently. One reviewer (NB) screened all, with two reviewers (AAA or RAS) each screening half. All papers deemed relevant by at least one reviewer were included in full-text screening. Full texts were also screened independently by at least two reviewers (NB and AAA or RAS). Any disagreements that arose between the reviewers at each stage of the study selection process were resolved through discussion with the review team (NB, AAA and RAS).

## Data charting

We developed the data charting template a priori informed by two reviews [47, 49], pilot tested it to refine its structure and content and iteratively refined it during the first part of the data charting process. For the final extraction, one reviewer (NB) extracted data from all studies, and a second reviewer (AAA) verified the extracted data for all studies with disagreements resolved through discussion and resolved by third reviewer (RAS). Data extracted included study characteristics (e.g., authors, year, country, pain management approach, study sample, targeted population), methodological components (e.g., cultural tailoring methods based on Leung et al. [49], theoretical frameworks, adapted content based on Stirman et al. [50]), and evaluation details (e.g., assessment type, primary or secondary outcomes) (see Appendix 3 for the final data extraction template).

We synthesised results narratively according to the study objectives and presented them in tables where appropriate. Categories for data presentation included both those predefined in our data extraction tool and those identified directly from the included studies. These categories encompassed the type of pain management approach, delivery format, origin or source of the approach, and the level of targeted population (i.e., minority population within a country, whole population in another country). To identify and categorise tailoring processes, we drew on the six-step adaptation framework developed by Leung et al. [49]. The six steps included: (a) information gathering, (b) preliminary adaptation design, (c) preliminary adaptation testing, (d) adaptation refinements, (e) adaptation trial, and (f) dissemination. This framework provided a structured approach to understanding how approaches were adapted for different populations or contexts. Additionally, we used the Framework for Reporting Adaptations and Modifications-Enhanced (FRAME) by Stirman et al. [50] to analyse and report on specific adaptation aspects, including: (a) when and how adaptations occurred during implementation, (b) whether the adaptation was planned or unplanned, (c) who decided on the adaptation, (d) which components were adapted, (e) the level of delivery at which the adaptation occurred, (f) whether the adaptation addressed context or content, (g) the extent of fidelity to the original programme, and (h) the rationale for the change. Elements of cultural adaptation aligned with FRAME could involve tailoring intervention *content,* such as materials, messages, or activities to reflect the target population’s cultural beliefs and values, using relevant examples or stories. The *context* may be modified to create a more culturally sensitive environment, including changes to physical settings or incorporation of cultural traditions. Adaptations can also affect *delivery* methods, adjusting communication styles to fit the preferences and norms of the population. *Training and evaluation* processes may be adapted to ensure staff cultural competence and to make assessment methods culturally appropriate. Finally, *implementation and scale-up* efforts often consider cultural factors by engaging community leaders, partnering with cultural organisations, and tailoring implementation strategies to the local cultural context [50]

## Results

**Fig. 1:**
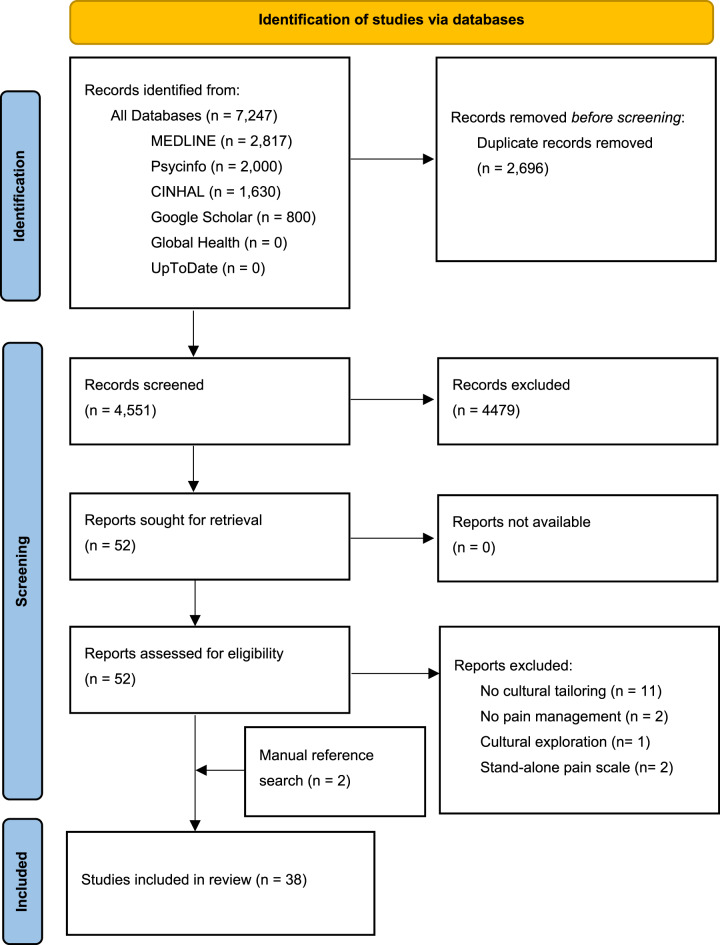
PRISMA flow chart of screening process

Figure 1 shows that the systematic search across the four databases identified 7,247 studies, of which 38 were included for data extraction and synthesis. Fourteen full texts were excluded because they did not provide information on cultural tailoring or did not focus on pain management.

## Study characteristics

Table 2 summarises the characteristics of the 38 included studies. The majority (*n* = 32; 84%) were published after 2016 and were conducted in North America (i.e., USA and Canada) (*n* = 18; 47%) with a focus on racial and ethnic minorities. Musculoskeletal pain was the primary focus in 26 (68%) studies, and 16 (42%) had a sample size between 10 and 50 participants.

**Table 2:** Descriptive summaries of publication year, study purpose, content, pain condition, and sample size across included studies (*n* = 38)

Study Characteristics		n (%)
Year of publication	2025–2021	16 (42%)
	2020–2016	16 (42%)
	2015–2010	5 (13%)
	2009–2000	1 (3%)
Study purpose	Inform/design and evaluate a tailored approach	18 (47%)
	Only inform/design a tailored approach	11 (24%)
	Only evaluate a tailored approach	9 (29%)
Geographical location	North America	18 (47%)
	Europe	6 (16%)
	Oceania	6 (16%)
	Africa	4 (11%)
	Asia	3 (8%)
	South America	1 (3%)
Health Condition	Musculoskeletal pain	26 (68%)
	Non-specific chronic pain	8 (21%)
	Cancer pain	4 (11%)
Sample size	10–50	16 (42%)
	> 100	11 (29%)
	50–100	5 (13%)
	< 10	3 (8%)
	Not reported	3 (8%)

## Tailored pain management approaches, target populations, and tailoring methods

### Approaches and populations

The 38 studies included in this review explored the cultural tailoring of 27 unique pain management approaches including education, self-management, and physiotherapy (see Table 3; Appendix 4 contains further approach-level details). Educational approaches were most common (*n* = 9; 33%), with 22 (81%) of the approaches (partly) delivered in-person. Thirteen (48%) of the approaches were newly developed, drawing on existing literature. These approaches primarily targeted specific minority populations within countries (*n* = 19; 70%). Content was the most commonly adapted element, (*n* = 25; 93%), followed by context (*n* = 14; 52%) and delivery (*n* = 12; 44%). Common cultural adaptations to content included adjustments to language (e.g., Brady [61]), the use of culturally relevant metaphors (e.g., Mukhtar et al. [62]), gender-specific considerations (e.g., Sleptsova et al. [63]), and the integration of local beliefs (e.g., Bezerra et al. [64]). One approach lacked information regarding the adaptation [65], while another was still in the planning stage of preliminary adaptation design [66].

**Table 3:** Descriptive summaries of pain management approaches (type, delivery format, source, target population), how these were tailored (which elements, use of frameworks, tailoring steps), and if tailoring was evaluated (*n* = 27* unique pain management approaches)

Type of pain management approach	Educational	9 (33%)
	Self-management programme	8 (30%)
	Cognitive behavioural therapy	5 (19%)
	Physiotherapy	5 (19%)
Delivery format	In-person/paper	22 (81%)
	Digital	4 (15%)
	Hybrid	1(4%)
Source/origin of the approach	Newly developed based on literature review	13 (48%)
	Existing approach from another country	7 (26%)
	Current guidelines in a country	3 (11%)
	Existing approach from the same country	3 (11%)
	Unclear	1 (4%)
Level of targeted population	Minority population within a country	19 (70%)
	Whole population in another country	8 (30%)
Elements of the approach that were tailored [50]**	Content (i.e. language)	25 (93%)
	Context (i.e. culture, setting)	14 (52%)
	Delivery	12 (44%)
	Training and Evaluation	2 (7%)
	Implementation and Scale-up	2 (7%)
	Not reported	2 (7%)
Explicit use of model/framework	Not reported	21 (78%)
	Yes^a^	6 (22%)
Steps of tailoring [49]**	Step 1: Information gathering	23 (85%)
	Step 2: Preliminary adaptation design	25 (93%)
	Step 3: Preliminary adaptation testing	17 (63%)
	Step 4: Adaptation refinement	3 (11%)
	Step 5: Cultural adaptation trial	11 (41%)
	Step 6: Dissemination	1 (4%)
Cultural tailoring evaluated?	Yes	22 (81%)
	No	5 (19%)

^*^ Total n represents the 27 unique pain management approaches described in the included 38 papers

^**^ Categories are not mutually exclusive

^a^ Model/framework used: Intervention Mapping-Adapt [51], FRAME [50], and ADAPT-IT [52]; Strengthening Families Programme model [53]; Planned Adaptation model [54]; Cultural security framework [55]; Bernal et al. [56]; Barrera & Castro [57]; Williams [58]; Card et al. [59]; Werner et al. [60]

Only four approaches were being delivered exclusively through digital formats. Of these, one was SMS-based [67], and three were internet-based [68–71]. Cultural tailoring of digitally delivered approaches primarily focused on content and context, incorporating culturally appropriate language, metaphors, and imagery. For example, Shayo et al. [67] employed tailoring strategies such as personalising text messages and coaching to align better with local needs. Similarly, Perry et al. [68] utilised culturally relevant multimedia content, including videos and animations featuring Māori imagery, language, and cultural concepts. This approach was designed in collaboration with Māori stakeholders, including cultural advisors, elders, and community members, to ensure the effective delivery of pain management strategies.

Of the 27 approaches, 19 were tailored to minority populations, mostly (*n* = 18) within high-income countries [61, 63, 66, 68–88]. For instance, Allen et al. [87] culturally adapted a pain coping skills training programme for African Americans with knee and hip osteoarthritis in the United States. This adaptation was based on previous clinical trials of pain coping skills among patients with chronic pain conditions. Similarly, Brady et al. [72] adapted a 12-week physiotherapy programme for three culturally and linguistically diverse communities in Australia. This adaptation drew on evidence-based guidelines for chronic pain management and aimed to enhance patient functioning through cognitive behavioural approaches. The programme included bilingual educators and culturally relevant content, developed through qualitative research, clinical expertise, and literature reviews.

The degree of content tailoring varied across approaches. While the majority (93%) tailored content, the extent and combination of adaptations differed. Six approaches focused solely on content [64, 70, 80–82, 84, 89], five combined adaptations to both content and context [62, 71, 75–77, 85, 90, 91], another six addressed content, context, and delivery [61, 63, 67, 72–74, 78, 83], while four tailored only content alongside delivery [79, 87, 92, 93]. Two approaches further extended adaptations to the implementation and scale-up phases [68, 69, 88]. For example, Perry et al. [68] collaborated with Māori community health trusts, researchers, and community members in co-designing and adapting the programme to enhance the potential for successful implementation and scale-up within these communities. In addition, two other approaches incorporated adaptations to training and evaluation components [86, 94, 95]. For example, training included active learning strategies such as role-playing, case studies, and group discussions to enhance facilitator skills and maintain fidelity of the approach. It also emphasised ongoing consultation and booster sessions to support facilitators and address challenges during implementation.

### Tailoring methods

As shown in Table 3, the included approaches mostly involved information gathering (*n* = 23, 85%) and preliminary adaptation design (*n* = 25, 93%) but rarely incorporated a refinement (*n* = 3, 11%) or dissemination (*n* = 1, 4%) phase.

Information gathering (step 1 from Leung et al. [49]), was reported in 23 studies (85%) and typically involved qualitative data collection to explore and assess cultural influences. This process often engaged patients, community members, and professionals, with varying levels of user involvement in the tailoring process. For example, Shayo et al. [67] interviewed expert clinicians complemented with literature reviews, while others, such as Duarte et al. [95] and Bezerra et al. [64], engaged patients and community members. Perry et al. [68] adopted a co-design approach, collaborating with both patient advisory group members and a technology design team. Common steps employed alongside information gathering were step 2 (preliminary adaptation design) and step 3 (preliminary adaptation testing). For example, Brady et al. [47, 60] applied a culturally tailored approach by adapting the content, context, and delivery [61] based on the guidelines proposed by Bernal et al. [56] and Barrera & Castro [57], and then preliminarily evaluated the adapted approach in a pilot randomized controlled trial [72].

While comprehensive tailoring efforts were rarely implemented, Hölzel et al. [81, 82] followed a more extensive tailoring process, addressing tailoring steps 1 to 5. This involved pilot testing with two individuals from each of four migrant groups in Germany (Turkish, Polish, Russian, and Italian), after which the materials were modified based on participant feedback. The refined, culturally tailored approach was then evaluated in a randomized controlled trial.

## Models and frameworks for guiding cultural tailoring

Models and frameworks, such as Intervention Mapping-Adapt [51], FRAME [50], and ADAPT-IT [52], guided the tailoring of six (22%) approaches. These framework-informed tailoring efforts were distinct from others by having a more systematic tailoring process, deeper and more iterative stakeholder engagement, more comprehensive cultural tailoring beyond language translation, and greater transparency in documenting adaptation decisions. In contrast, studies without a formal framework often used ad hoc methods, limited or one-time stakeholder input, and provided minimal detail on the adaptation process. For example, Yin et al. [79] implemented a chronic pain self-management program in a rural community with limited stakeholder engagement and minimal documentation of adaptation decisions, while Pagán-Ortiz & Cortés [70] delivered a digital intervention for Latinas with chronic pain without a detailed description of how cultural adaptations were made or justified.

Moreover, while some employed a mostly linear process, others were more flexible and iterative. For example, Brady et al. [61] described distinct phases -such as information gathering, content adaptation, and pilot testing- but emphasised the dynamic, non-linear interplay between these stages. Similarly, Duarte et al. [95], employed a structured approach with defined steps, including team training, materials translation, needs assessments through focus groups and expert interviews, instructor training, and pilot testing to ensure the programme’s relevance and effectiveness. However, while following a clear structure, the team made adjustments throughout the process, incorporating participant feedback and cultural considerations to refine the pain management approach. They also used multiple frameworks to guide adaptation, ultimately streamlining the process into a linear model for the Portuguese cultural adaptation of the Fit & Strong programme from the USA original version. In contrast, Monroe et al. [94], followed a clear iterative process, employing the Intervention Mapping-Adapt and FRAME frameworks to adapt an existing psychologically informed physical therapy intervention. This six-step iterative process led to the development of the goal-oriented activity intervention for Hispanic populations in the United States.

## Evaluation of cultural tailoring impact

Among the reviewed approaches, more than half (*n* = 17, 63%) were preliminarily tested using pilot or feasibility studies. For example, Duarte et al. [95] conducted a pilot study that evaluated the acceptability of the intervention through qualitative methods. This included session observations to identify implementation challenges and areas for improvement, as well as participant interviews to assess the programme’s acceptability, rather than quantitatively measuring its effectiveness. Eleven studies (41%) more fully evaluated the outcomes of their culturally tailored programmes, with seven using randomized controlled trials to assess effectiveness. The primary outcomes measured in these randomized controlled trials centred on self-efficacy [63, 84], patient-rated usefulness [82], symptom management knowledge, and quality of life [77]. These outcomes were predominantly assessed immediately following the intervention, with limited attention given to longer-term impacts. Only two studies conducted follow-up assessments extending beyond six months, one at nine months [87] and another at twelve months [63]. Of the included RCTs, three compared the tailored approach with usual care [77, 82, 87], while one other compared two culturally sensitive approaches [63]). One study compared three groups: a control group, a translated version of the approach, and a culturally sensitive version [90] (Appendix 4 contains further approach-level details). The studies generally reported that culturally tailored educational and psychosocial interventions led to short-term improvements in pain knowledge and coping compared to non-tailored versions, with good participant adherence and satisfaction.

## Discussion

This scoping review explored cultural tailoring of pain management approaches. We included 38 studies, describing the tailoring of 27 unique pain management approaches. Most studies were published after 2016, focusing on culturally tailored pain management for racial and ethnic minorities living with chronic musculoskeletal pain in high income countries. Educational approaches were most commonly tailored, adapting content through culturally relevant language, metaphors, and gender considerations. Most studies conducted information gathering and preliminary adaptation design as part of the tailoring process, although only six were guided by established frameworks. The effectiveness of cultural tailoring was assessed for the majority of approaches, primarily through pilot studies and randomised controlled trials. This trend suggests increasing attention to culturally tailored pain management but also highlights the need for broader inclusion of diverse populations and settings globally.

## Relation to other studies

The findings of this review aligned with prior research on cultural tailoring of healthcare interventions, while also uncovering distinct gaps and areas for improvement in the context of in pain management. Previous reviews on the cross-cultural tailoring of pain self-assessment tools showed that the extent and evaluation of cultural tailoring varied across studies [33–39]. We found that tailoring of pain management approaches generally extended beyond translation to include culturally relevant language, metaphors, and gender considerations (e.g. Mukhtar et al. [62]; Sleptsova et al. [63]) but often remained content-focused, with limited attention paid to contextual changes, implementation strategies, and training. This suggests that, although progress has been made in the cultural tailoring of pain assessment and management approaches, there remains a need for more comprehensive tailoring efforts. While cultural tailoring is evolving from basic linguistic translation to more nuanced content adaptations, it still falls short of addressing the broader cultural and systemic factors that shape pain experience and management. This is especially critical in pain management, where tailoring should consider cultural norms around pain expression, stoicism, and help-seeking behaviours, in addition to general adaptations such as language and metaphors [19].

We identified very few culturally tailored digital pain management approaches, despite their increasing use in healthcare [96]. In contrast, digital approaches for managing mental health have seen more extensive cultural tailoring; Spanhel et al. [29] identified 55 culturally adapted digital interventions in this domain. Those identified in this review used interactive and multimedia features (e.g., Pagán-Ortiz & Cortés [70]) while a smaller number employed simpler formats such as short message service (SMS). These SMS-based approaches offered accessible and scalable solutions that were well-suited to the technological environments in which they were implemented (e.g., Shayo et al. [67]). In comparison, culturally tailored digital interventions for mental health often made greater use of interactive functionalities such as enhancing user engagement, simplifying navigation to improve user experience, and adapting visuals to align with local cultural sensibilities [29], highlighting a notable contrast in the level of cultural tailoring between digital approaches for pain management and mental health. However, digital approaches require careful consideration of access, affordability, and cultural acceptability to ensure they effectively reach the intended populations.

Although we found that most studies engaged in initial steps like information gathering and adaptation design, the use of established cultural adaptation frameworks varied widely. Compared with Barrera et al.’s [23] staged model, Day et al.’s [24] synthesis, and Costas-Muñiz et al.’s [25] practical guide, pain management approaches largely implemented early, surface-level adaptations, such as content and language modification. However, relatively few studies progressed to the deeper stages recommended by these frameworks, such as pilot testing, iterative stakeholder feedback, or evaluation of cultural fit. This is consistent with Leung et al. [49], the review found that cultural adaptation processes in public health interventions were often not systematic, which may contribute to inconsistencies in adaptation processes and outcomes. Similarly, Joo & Liu [22] identified weaknesses in culturally tailored interventions for ethnic minorities with chronic illnesses, including unclear guidelines and insufficient training. These parallels highlight persistent gaps in the comprehensive and structured application of cultural adaptation across different health domains.

## Limitations

Several limitations should be considered when interpreting the findings of this review. Although our search strategy was comprehensive, the strategy may not have captured all relevant studies. For example, those that used related terminology relevant to cultural tailoring not included in our search syntax, such as terms related to ethnicity, race, or language. This means we may have missed some relevant cultural tailoring efforts.

Additionally, this review included only studies published in English. This means we did not include initiatives published in other languages, which had a potential negative impact on the comprehensiveness of our review, in particular regarding global cultural tailoring efforts across diverse linguistic and cultural contexts. Lastly, we did not include a critical appraisal of the quality of included studies. Although this is not requirement for scoping reviews, it would have provided useful insights into the need for higher quality research on cultural tailoring. However, the design and focus of our review does not allow us to draw any conclusions on this.

## Implications for practice and research

Researchers should prioritise culturally sensitive digital pain management approaches, given the increased use of digital technologies in pain management. Despite the potential of digital formats to enhance accessibility and effectiveness of pain management approaches for diverse populations, we only found few examples of this, many of which failed to fully harness the approaches’ digital nature. Insights from mental health research, where cultural tailoring of digital interventions is more advanced, could inform future efforts to culturally tailor digital pain management approaches that better leverage interactive capabilities [29, 30].

Policymakers, researchers, and healthcare practitioners must collaborate to improve the design, implementation, and evaluation of culturally tailored pain management approaches. Initiatives to date focussed on content modifications alone, with insufficient attention to contextual changes, provider training, and structured implementation strategies. As advocated by Naderbagi et al. [47], a more comprehensive approach that integrates these elements and involves a wider range of stakeholders could enhance effectiveness, as shown by Perry et al. [68] and Brady et al. [72]. Applying established frameworks (i.e. [23–25]) can help maintain intervention effectiveness while adapting to cultural nuances, ensuring interventions are both culturally relevant and evidence-based. Similarly, cultural tailoring should move beyond ethnic minorities in high-income countries to address broader systemic and contextual differences in countries of origin and low- and middle-income countries. For example, Aldosari et al. [97] found that digital health interventions for South Asians in high-income countries may not apply in South Asian nations due to variations in sociocultural contexts, healthcare systems, and technology access. Future research should consider these differences to ensure that culturally tailored pain management approaches are effective more broadly across countries.

Finally, future research should incorporate alternative evaluation methods, such as feasibility, acceptability, and usability studies, to better understand how culturally tailored pain management approaches work in real-world settings. Current evaluations focus largely on effectiveness, but broader healthcare outcomes such as patient satisfaction and access should also be considered [98]. For instance, Duarte et al. [95] demonstrated the value of such evaluations in real-world contexts, as well as demonstrated by Lin et al. [99]. Adopting these methods will provide deeper insights into the practical implementation of culturally tailored approaches, helping healthcare providers and policymakers to optimise pain management for diverse populations.

## Conclusion

This review identified several efforts to culturally tailor pain management approaches, particularly for racial and ethnic minorities with musculoskeletal pain living in high income countries. While content adaptations of in-person and paper-based approaches were common, other aspects such as contextual modifications and tailoring, especially of digital delivery formats, were under-researched. Randomised controlled trials often evaluated the impact of culturally tailored approaches on efficacy, knowledge and quality of life, whereas qualitative evaluations of other outcomes appeared scarce. Future research should prioritise the cultural tailoring of digital approaches and extend tailoring efforts beyond content modification. Employing more diverse evaluation methods will be essential for understanding the feasibility, acceptability and real-world effectiveness of culturally tailored pain management approaches for more equitable pain services and outcomes.

## Electronic supplementary material

Below is the link to the electronic supplementary material.

Supplementary Material 1

## Data Availability

Data is provided within the manuscript or supplementary information files.
