# Supplementary material for: Cultural tailoring of pain management approaches: a scoping review
Source: Int J Equity Health. 2025 Dec 20;24:354. doi: 10.1186/s12939-025-02743-5 (PMC12752237; doi:10.1186/s12939-025-02743-5)
Supplement: Supplementary file 1 — Supplementary Material 1 [file 12939_2025_2743_MOESM1_ESM.docx]

**Appendices**

**Appendix 1 Preferred Reporting Items for Systematic reviews and Meta-Analyses extension for Scoping Reviews (PRISMA-ScR) Checklist**

| **SECTION** | **ITEM** | **PRISMA-ScR CHECKLIST ITEM** | **REPORTED ON PAGE #** |
| --- | --- | --- | --- |
| **TITLE** | | | |
| Title | 1 | Identify the report as a scoping review. | 1 |
| **ABSTRACT** | | | |
| Structured summary | 2 | Provide a structured summary that includes (as applicable): background, objectives, eligibility criteria, sources of evidence, charting methods, results, and conclusions that relate to the review questions and objectives. | 2 |
| **INTRODUCTION** | | | |
| Rationale | 3 | Describe the rationale for the review in the context of what is already known. Explain why the review questions/objectives lend themselves to a scoping review approach. | 5 |
| Objectives | 4 | Provide an explicit statement of the questions and objectives being addressed with reference to their key elements (e.g., population or participants, concepts, and context) or other relevant key elements used to conceptualize the review questions and/or objectives. | 6 |
| **METHODS** | | | |
| Protocol and registration | 5 | Indicate whether a review protocol exists; state if and where it can be accessed (e.g., a Web address); and if available, provide registration information, including the registration number. | 6 |
| Eligibility criteria | 6 | Specify characteristics of the sources of evidence used as eligibility criteria (e.g., years considered, language, and publication status), and provide a rationale. | 6 |
| Information sources | 7 | Describe all information sources in the search (e.g., databases with dates of coverage and contact with authors to identify additional sources), as well as the date the most recent search was executed. | 9 |
| Search | 8 | Present the full electronic search strategy for at least 1 database, including any limits used, such that it could be repeated. | 40 |
| Selection of sources of evidence | 9 | State the process for selecting sources of evidence (i.e., screening and eligibility) included in the scoping review. | 9 |
| Data charting process | 10 | Describe the methods of charting data from the included sources of evidence (e.g., calibrated forms or forms that have been tested by the team before their use, and whether data charting was done independently or in duplicate) and any processes for obtaining and confirming data from investigators. | 9 |
| Data items | 11 | List and define all variables for which data were sought and any assumptions and simplifications made. | 41 |
| Critical appraisal of individual sources of evidence | 12 | If done, provide a rationale for conducting a critical appraisal of included sources of evidence; describe the methods used and how this information was used in any data synthesis (if appropriate). | N/A |
| Synthesis of results | 13 | Describe the methods of handling and summarizing the data that were charted. | 10 |
| **RESULTS** | | | |
| Selection of sources of evidence | 14 | Give numbers of sources of evidence screened, assessed for eligibility, and included in the review, with reasons for exclusions at each stage, ideally using a flow diagram. | 11 |
| Characteristics of sources of evidence | 15 | For each source of evidence, present characteristics for which data were charted and provide the citations. | 12 |
| Critical appraisal within sources of evidence | 16 | If done, present data on critical appraisal of included sources of evidence (see item 12). | N/A |
| Results of individual sources of evidence | 17 | For each included source of evidence, present the relevant data that were charted that relate to the review questions and objectives. | 41 |
| Synthesis of results | 18 | Summarize and/or present the charting results as they relate to the review questions and objectives. | 11-20 |
| **DISCUSSION** | | | |
| Summary of evidence | 19 | Summarize the main results (including an overview of concepts, themes, and types of evidence available), link to the review questions and objectives, and consider the relevance to key groups. | 20 |
| Limitations | 20 | Discuss the limitations of the scoping review process. | 22 |
| Conclusions | 21 | Provide a general interpretation of the results with respect to the review questions and objectives, as well as potential implications and/or next steps. | 24 |
| **FUNDING** | | | |
| Funding | 22 | Describe sources of funding for the included sources of evidence, as well as sources of funding for the scoping review. Describe the role of the funders of the scoping review. | 25 |

**Appendix 2 Search conducted on MEDLINE (Ovid) on 13 September 2024**

|  | Search terms | Results |
| --- | --- | --- |
| 1. | "sociocultural factors".mp. | 2263 |
| 2. | (cultural* adj3 sensitiv*).mp. [mp=title, book title, abstract, original title, name of substance word, subject heading word, floating sub-heading word, keyword heading word, organism supplementary concept word, protocol supplementary concept word, rare disease supplementary concept word, unique identifier, synonyms, population supplementary concept word, anatomy supplementary concept word] | 7799 |
| 3. | (cultural* adj3 adapt*).mp. [mp=title, book title, abstract, original title, name of substance word, subject heading word, floating sub-heading word, keyword heading word, organism supplementary concept word, protocol supplementary concept word, rare disease supplementary concept word, unique identifier, synonyms, population supplementary concept word, anatomy supplementary concept word] | 11013 |
| 4. | (language adj3 translat*).mp. [mp=title, book title, abstract, original title, name of substance word, subject heading word, floating sub-heading word, keyword heading word, organism supplementary concept word, protocol supplementary concept word, rare disease supplementary concept word, unique identifier, synonyms, population supplementary concept word, anatomy supplementary concept word] | 1544 |
| 5. | (local adj3 context).mp. [mp=title, book title, abstract, original title, name of substance word, subject heading word, floating sub-heading word, keyword heading word, organism supplementary concept word, protocol supplementary concept word, rare disease supplementary concept word, unique identifier, synonyms, population supplementary concept word, anatomy supplementary concept word] | 4593 |
| 6. | (cultur* adj3 appropriate*).mp. [mp=title, book title, abstract, original title, name of substance word, subject heading word, floating sub-heading word, keyword heading word, organism supplementary concept word, protocol supplementary concept word, rare disease supplementary concept word, unique identifier, synonyms, population supplementary concept word, anatomy supplementary concept word] | 9948 |
| 7. | cross cultur*.mp. | 40126 |
| 8. | (cultural* adj3 tailor*).mp. | 2675 |
| 9. | (cultural* adj3 safe*).mp. | 1903 |
| 10. | (cultural* adj responsiv*).mp. | 1336 |
| 11. | (cultur* adj3 adjust*).mp. | 824 |
| 12. | (cultur* adj3 modif*).mp. | 4412 |
| 13. | 1 or 2 or 3 or 4 or 5 or 6 or 7 or 8 or 9 or 10 or 11 or 12 | 78580 |
| 14. | pain.mp. | 931347 |
| 15. | 13 and 14 | 2964 |
| 16. | Limit 15 to English language | 2817 |

**Appendix 3 Data Charting template**

| **Objective 1 Study characteristics** | | | | | | | **Objective 2 Method of adaptation** | | | | | | **Objective 3 Impact evaluation** | | |
| --- | --- | --- | --- | --- | --- | --- | --- | --- | --- | --- | --- | --- | --- | --- | --- |
| **Author (Year)** | **Region/Country** | **Condition** | **Study Aim** | **Study Design** | **Study Sample (Size/Type)** | **Approach** | **Source/Origin of Intervention** | **Models/ Theoretical Approach** | **Process** | **Adapted Content** | **Elements of tailoring** | **Tailoring Steps** | **Impact (Yes/No)** | **If Yes, Assessment Type** | **Findings /Outcomes** |
|  |  |  |  |  |  |  |  |  |  |  | Elements of tailoring according to Stirman et al. (50):  Context  Content  Delivery  Training and Evaluation  Implementation and Scale-up | Steps according to Leung et al. (49). Step 1: Info gathering, Step 2: Prelim. Adapt. Design, Step 3: Prelim. adapt. Testing, Step 4: Adapt. Refinement, Step 5: Cult. Adapt. Trial, Step 6:  Dissemination |  |  |  |

**Appendix 4 Overview of Culturally Tailored Pain Management approaches (n=27)**

| **Reports** | **Condition** | **Target Population** | **Approaches** | **Adapted Content** | **Models and Frameworks** | **Tailoring steps^a^** | **Description of evaluation** |
| --- | --- | --- | --- | --- | --- | --- | --- |
| **Non-digital and hybrid approaches** | | | | | | | |
| Brady et al. (51), (72) | Chronic musculoskeletal pain | Three communities (Mandaean, Assyrian and  Vietnamese) in Australia | 12-week physiotherapy pain management approach (included a combination of education and exercise) | Language, persons (culturally sensitive bilingual health educator), metaphors, content, concept, goals, methods of delivery, and context. | Bernal et al.  (62); Barrera & Castro (63) | 1,2,3 | Prospective, assessor-blinded, multicentre pilot RCT comparing culturally adapted physiotherapy to standard care. Primary outcomes were feasibility of and engagement of the tailored approach, assessed through attendance, adherence, and satisfaction. |
| Sleptsova et al. (53) | Chronic pain | First-generation Turkish immigrants residing in Switzerland | cognitive behavioural treatment | Include gender-separated groups, extended number of sessions (14 to 25), Turkish sessions with an interpreter, minimal written materials for illiterate patients, and graphical aids to explain exercises and the emotion-tension-pain cycle | Not reported | 5 | RCT comparing culturally sensitive cognitive behavioural therapy to culturally sensitive exercise therapy. Primary outcome was efficacy of the culturally  sensitive cognitive-behavioural treatment, on Physical Functioning and Mental Health, with 12 months follow-up. |
| Mukhtar et al., (52), (90) | Chronic neck pain | Hausa speaking patients in Nigeria | Pain Neuroscience Education | Materials were simplified, audio-based, and tailored for content, language, and delivery method. They included gender-specific resources, cultural metaphors and visuals, and home education audio materials. | Not reported | 1,2,3 | Pilot RCT evaluating recruitment, feasibility, and preliminary effectiveness of the culturally tailored approach by comparing translated standard Pain Neuroscience Education (PNE), culturally sensitive PNE, and an Exercise Therapy control group. Primary outcomes were pain intensity, and disability. |
| Bezerra et al. (54) | Neck pain | Brazilian patients | Four‐week Home Exercise Protocols – adapted from literature. | Tailored to local practices and beliefs. Focusing on exercise clarity, ease of execution, time commitment, 4-week suitability, and pamphlet format | Not reported | 1,2,3,4 | Observational study with cultural adaptation via sessions with target users. Feedback on clarity, usability, motivation, and format collected at 2 and 4 weeks. Pain intensity and adherence monitored. |
| Duarte et al. (95) ^b^ | Osteoarthritis | Portuguese patients | Fit & Strong! A physical activity/behaviour change evidence-based intervention for persons with osteoarthritis – adapted from American original intervention. | Format and content to increase its cultural appropriateness | ADAPT-IT (58); Strengthening Families Programme model (59); and Planned Adaptation model (60); | 1,2,3,4 | Pilot study assessing acceptability of the pain management approach through observations, participant interviews, and instructor questionnaires. |
| Der Ananian et al. (101) ^b^ | Arthritis | Spanish-speaking Hispanics in USA | Fit & Strong! Group-based exercise program delivered in Spanish by bilingual, bicultural instructors | Language (Spanish manual), delivery approach | Not reported | 2,5 | Single-group pre-post design assessing implementation feasibility and the tailored approach effectiveness on lower-extremity strength, aerobic endurance, perceived pain, physical function and stiffness, self-efficacy for arthritis management, and self-efficacy for exercise, with follow-up for all outcome measure at 4 months post intervention. |
| Hodge et al. (76), (75), (77) | Cancer pain | American Indian in USA | “Weaving Balance into Life” toolkit: booklet, video, and resource guide | Toolkit content co-developed with community input; integrated imagery, language, colour schemes, and themes (spirituality, balance, storytelling) | Not reported | 1,2,5,6 | Hodge et al. (75) pre-post-test design comparing a tailored toolkit via talking circles to standard care. The primary outcome was self-reported pain management ability at 6 months.  Hodge et al. (77) RCT comparing a culturally tailored toolkit intervention to a control (dental care education) with pre-post measures. Primary outcomes included symptom management knowledge and behaviour, and quality of life. |
| Phaneth et al. (91) | Chronic pain | Cambodian adults | “Pain school” a ten-session, group-based, interdisciplinary pain education intervention – adapted from original Danish intervention | tailored to local cultural norms, work practices, and social support systems (e.g., Buddhist temples instead of welfare state) | Not reported | 1,2,3 | Pilot pre-post study using quantitative and qualitative measures to assess the tailored approach clinical improvements in pain and function, alongside therapist focus groups and Strengths, Weaknesses, Opportunities, and Threats analysis to evaluate the programme’s relevance, helpfulness, and implementation factors. |
| Corsino et al. (78) | Knee and Hip Osteoarthritis | Spanish-speaking adults in USA | telephone-delivered intervention focused on physical activity, weight management, and cognitive-behavioural pain management skills | Materials adapted to reflect cultural/language diversity within Hispanic/Latino populations; included region-specific Spanish language, culturally relevant photos, and local organization expertise in content development | Not reported | 1,2,3 | Feasibility pilot study assessing recruitment, intervention delivery, as well as the tailored approach effect on pain, stiffness, and function. |
| Davis et al. (89) | Hip/Knee Osteoarthritis | Canadian adults | (GLA:D^TM^) two education sessions and 12 supervised, neuromuscular exercise classes – adapted original Danish intervention | GLA:D program translated from Danish to Canadian English; revised language, terminology, grade level, and added local epidemiological data. Therapist training localized with Canadian context and standards. | Not reported | 1,2,5 | Mixed-methods feasibility study using pre-post surveys, class observation, and therapist interviews to assess the tailored intervention in the Canadian context with 3 months follow-up to evaluate Reach, Effectiveness and Maintenance. |
| Nkhata et al. (93) | Low back pain | Zambian nurses with Low back pain | A 12-week educational campaign based on evidence-based back pain messages | message format and delivery tailored to culture, nurse preferences and hospital environment | Not reported | 1,2,5 | Single-sample pre- and post-test design evaluating a 12-week cross-cultural back pain education campaign. Primary outcomes included beliefs about back pain and self-management activation. |
| Chen et al. (55) | Chronic pain | Chinese adults | 8-week Modified Mindfulness-Based Stress Reduction Programme | details of the modifications not described in the paper; adaptation outline reported only in supplementary materials; referred to as "culturally adaptive" but specifics are unclear | Not reported | 3 | Pilot RCT comparing a culturally modified Mindfulness-Based Stress Reduction Programme versus usual care. Assessing the tailored approach effect on pain severity, pain interference, depression, perceived stress, pain catastrophising, mindfulness, and resilience with 3-month follow-up for all measures as primary outcomes. |
| Sharma et al. (92) | Low back pain | Nepalese adults | Pain education –informed by Australian resources | Materials translated into Nepali; culturally adapted with stories from local patients/clinicians; visuals emphasized over text; family involvement for low literacy accommodation | Not reported | 1,2,3,4,5^c^ | Planned assessor-blinded feasibility RCT comparing culturally tailored pain education to guideline-based physiotherapy. Primary outcomes focused on trial feasibility. |
| Yin et al. (79) | Chronic pain (lower-body pain) | Predominantly older rural Latino adults in USA | "Living Better Beyond Pain" (LBBP) a six-week low-literacy low-cost patient-cantered self-management programme | Low-literacy, bilingual (English-Spanish) program, culturally adapted materials and instruction; | Not reported | 1,2,3 | Feasibility study using a one-group design, assessing the tailored approach feasibility and its impact on physical, cognitive, and psychosocial functioning using eight outcome measures with 3 months follow-up. |
| Allen et al. (87), Schrubbe et al.  (102) | Knee and hip OA | African Americans in USA | telephone-delivered culturally tailored pain Coping Skills Training telephone | Pain coping skills to be compatible with cultural, spiritual, religious, or other values.  Culturally tailored through input from African American patients, providers, and community members on relevance, clarity, and cultural fit. Counsellors connected skills with cultural or religious values using active listening. | Not reported | 1,2,5 | Multi-site RCT comparing culturally tailored CST to usual care. Primary outcome was Western Ontario and McMasters Universities Osteoarthritis Index pain subscale with 3 (primary) and 9 months follow-up. |
| Booker et al. (56) | Knee Osteoarthritis | African American/Black older adults | "PROACTIVE" a mechanism-based pain self-management intervention | Not yet implemented; planned to co-develop culturally tailored intervention via focus groups based on biopsychosocial–behavioural predictors | Not reported | 1,2 | No evaluation reported in this protocol; goal is to inform culturally tailored intervention development for later testing |
| Orhan et al. (74), (73) | Chronic low back pain | First-generation Turkish migrants in Belgium | Pain neuroscience education | Content adapted, examples, visual information (i.e. illustrations and pictures), and metaphors were adapted based on the gender- and culture-related characteristics | Not reported | 1,2,3 | Pilot RCT comparing culture-sensitive versus standard pain neuroscience education. Assessing the tailored approach effect on pain knowledge, pain intensity, and perceived disability as primary outcomes. |
| Moonaz et al. (80) | Chronic pain | Underserved adults with chronic pain at federally qualified health centres FQHCs (including Spanish-speakers) | Flexible Yoga Therapy Protocol | handouts translated to Spanish, back-translated, literacy-adapted, photo-illustrated; bilingual delivery with interpreter support available; unique handout sets for each clinic | Not reported | 2,3 | Quasi-experimental pilot combining yoga therapy and acupuncture. Feasibility evaluated through fidelity checks, individual charting, and provider consistency. No formal clinical outcomes reported. |
| Hölzel et al. (82), (81) | non-specific chronic low back pain | Adults with Turkish, Polish, Russian, or Italian migration background living in Germany | Culturally sensitive patient information materials (PIMs) | final brochures included visual and narrative changes for cultural appropriateness | Not reported | 1,2,3,4,5 | Multicentre, double-blind RCT comparing culturally sensitive patient information materials to standard translated materials. The primary outcome was patient-rated usefulness, assessed immediately after receiving the material with 8 weeks and  6 months follow-up as secondary outcome. |
| Cornelio-Flores et al. (83) | Chronic pain | Spanish-speaking Latinos in USA | Integrative Medical Group Visit (IMGV) curriculum | Hispanic poetry, culturally relevant recipes, simplified language, and family-cantered framing; sessions led by Spanish-speaking clinicians | Not reported | 1,2,3 | Pre–post pilot feasibility study to assess the tailored approach. using quantitative assessment to assess pain impact, depressive  symptoms, anxiety, and perceived stress pre and post intervention; and qualitative focus groups and interviews to explore satisfaction, cultural relevance, and acceptability of the adaptation of the curriculum. |
| Swerissen et al. (84) | Chronic disease (arthritis) | Vietnamese, Chinese, Italian, and Greek Australians with chronic illness | Chronic Disease Self-Management Program (CDSMP) | CDSMP translated and culturally adapted (content, delivery, minor cultural adjustments). The program was taught by a pair of trained bilingual peer- leaders | Werner et al. (66) | 2,5 | RCT comparing CDSMP to waitlist control at 6 months. The primary outcome was health status, health behaviours, self-efficacy and health service utilisation. Data collection was completed at baseline and 6 months later. Health service utilisation was collected at monthly intervals for the 6 months. |
| Garza (85) | Somatic symptom disorder with predominant pain | Immigrant Mexican-American women | Outpatient 12-session psychotherapy intervention integrating cognitive behavioural therapy and acceptance and commitment therapy | Cultural beliefs: family, religion, fatalism | Not reported | 1,2,3^c^ | Planned pilot evaluation of the tailored approach effect on pain complaint levels, daily functioning levels and severity of depression, at scheduled intervals. |
| Lin et al. (88) | Low Back Pain | Aboriginal Australians in a rural area | “My Back on Track, My Future” Audiovisual resource for Low Back Pain information | Co-designed with Aboriginal communities. Focused on cultural relevance, appropriate language, traditional storytelling, preferred visual learning, and correcting five key low back pain misconceptions. | Cultural security framework (61) | 1, 2 | Qualitative randomized crossover study comparing culturally adapted and standard information (Back Book). Participants assessed the tailored approach clarity, relevance, and usability. |
| Gombatto et al. (86), Monroe, et al (94), (103) | Chronic musculoskeletal pain | Latino/Hispanic adults in Southwestern USA | Goal Oriented Activity (GOALS/Metas), An 8-week hybrid telerehabilitation program integrating cognitive behavioural therapy-based physical therapy | Context setting and format; Content and training modified to specific cultural and linguistic factors and lower health literacy.  culturally relevant content (e.g., addressing fatalism, promoting self-efficacy, integrating prayer and family support). | Intervention Mapping-Adapt (57); and FRAME (50), | 1,2,5^c^ | Gombatto et al. (86) protocol outlines A single-blind, 2-arm parallel group, superiority RCT comparing the tailored approach with usual care physiotherapy to assess the primary outcome of pain-related disability. The trial also includes follow-up assessments at 1 week, 3 months, and 6 months post-intervention. |
| **Digital approaches** | | | | | | |  |
| Shayo et al. (67) | Musculoskeletal associated pain and disability | Tanzanian patients | Evidence-based approaches to support home-based rehabilitation using telephone calls and SMS text messaging via mobile phones –adapted from American original intervention | Selected effective telephone coaching model and integrated evidence-based strategies for text messaging to meet needs. Culturally adapted text messages, culture appropriate local images and Swahili-language for handouts | Williams et al. (64); Card et al. (65) | 1,2 | No evaluation reported in this paper; focus was on describing the cultural and contextual adaptation process |
| Perry et al. (68),  Hale et al. (69) | Persistent pain | Māori (Indigenous population of Aotearoa) in New Zealand | iSelf-help, online group-based pain management programme | Welcome page to iSelf-help; whānau stories (family member and significant others); and videos and metaphor | Not reported | 1,2,5^c^ | Hale et al. (69) protocol for a pragmatic,  multicentred, assessor­ blinded, two­ arm, parallel group, non­ inferiority RCT comparing the tailored approach with an in-person delivered pain management programme. Assessing pain-related disability at 6 months post intervention as the primary outcome. |
| Pagán-Ortiz & Cortés (70) | Chronic primary/nonspecific pain. | Spanish-speaking Latinas with chronic pain in USA | Online, self-guided educational intervention with videos, text, and SMS reminders | Adapted from the evidence-based LAMP workbook. Translated into Spanish with cultural tailoring for Latina populations, formatted for mobile, and supplemented with video content. Reviewed by Latino health and chronic pain experts for cultural and clinical relevance. | Not reported | 1,2,3 | Mixed-method single-arm pilot study with pre-post design, to assess patients’ and providers’ acceptability of and satisfaction with the tailored intervention. As well as the impact of the tailored approach on patients’ knowledge, chronic pain self-efficacy,  pain severity and pain interference. |
| Chee et al. (71) | Cancer pain (Breast cancer) | Asian American breast cancer survivors (Chinese, Korean, Japanese) | Technology-based intervention Cancer Pain management support Program for Asian  American survivors of breast cancer, with discussion board, educational sessions, and online resources, delivered in four languages | Developed in four languages with culturally matched staff. Focused on culturally appropriate language, visuals, pain beliefs, relevant examples, moderated support, and daily responsive coaching. Included multimedia features and 20 culturally specific cancer pain resources | Not reported | 1,2,3 | Pilot RCT with repeated measures, comparing the tailored approach to a control groups (the American Cancer Society multilingual website on breast cancer and cancer pain management), assessing the primary outcome of cancer pain severity. |

*^RCT^* ^randomised controlled trial^

^aSteps according to Leung et al. (49): Step 1: Information gathering, Step 2: Preliminary adaptation design, Step 3: Preliminary adaptation testing, Step 4: Adaptation refinement, Step 5: Cultural adaptation trial, Step 6: Dissemination^

^bSame intervention but have been tailored in different country sitting and adaptation process^

^cProtocol/planned study^
